# A viscosity-induced voltage response microfluidic triboelectric sensor for real-time monitoring of blood coagulation

**DOI:** 10.1007/s00604-026-08044-0

**Published:** 2026-04-21

**Authors:** Jia-Cheng Lin, I-Chang Su, Yong-Kwang Tu, Ningappa Kumara Swamy, Kuang-Chong Wu, Horn-Jiunn Sheen, Yu-Jui Fan

**Affiliations:** 1 Institute of Applied Mechanics, National Taiwan University, 1 Roosevelt Rd., Sec. 4, Taipei, 10617 Taiwan; 2 Taipei Neuroscience Institute, Taipei Medical University, Taipei, 11031 Taiwan; 3 Department of Neurosurgery, Shuang Ho Hospital, Taipei Medical University, New Taipei City, 23561 Taiwan; 4 Department of chemistry, JSS Science and Technology University, Mysuru, 570006 India; 5 Department of Mechanical Engineering, National Yang Ming Chiao Tung University, Hsinchu, 300093 Taiwan

**Keywords:** Microfluidic mixing, Triboelectric nanogenerator (TENG), Blood coagulation time, Activated partial thromboplastin time (APTT), Prothrombin time (PT)

## Abstract

Blood coagulation is a critical physiological process, and its abnormal or unpredictable occurrence can cause severe medical complications in clinical settings. Rapid and accurate assessment of coagulation status is therefore essential, particularly in clinical trials where timely monitoring is crucial. We developed a microfluidic device integrating a micromixer and multi-signal acquisition system for real-time measurement of blood coagulation times. The device utilizes secondary flow generated at two curves of an expansion–contraction serpentine microchannel to mix plasma, reagents, and calcium ions efficiently. Voltage signals induced by the triboelectric effect between the flowing fluid and a copper wire electrode are collected continuously, enabling precise monitoring of changes in blood viscosity during clot formation. The proposed system requires only 20 µL of sample, achieves coagulation detection within 2 min, and shows measurement deviations of less than 5% compared to a commercial coagulation analyzer. By analyzing these voltage changes, both activated partial thromboplastin time (APTT) and prothrombin time (PT) can be determined accurately. The device is compact, low-cost, and portable, making it suitable for point-of-care applications and settings where rapid blood status evaluation is needed. Overall, this microfluidic coagulation chip offers a practical alternative to conventional laboratory assays, combining minimal sample consumption, fast detection, and reliable performance, thereby providing an effective tool for monitoring patient coagulation status in clinical trials and potentially in broader medical applications.

**Graphical Abstract:**

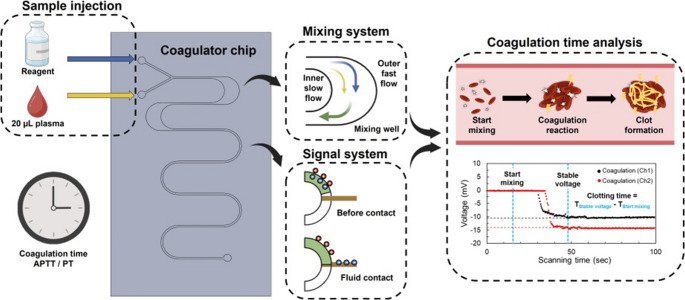

**Supplementary Information:**

The online version contains supplementary material available at 10.1007/s00604-026-08044-0.

## Introduction

Coagulation tests are essential for assessing patients at risk of hemorrhage, including those preparing for surgery or under postoperative care. Among available assays, activated partial thromboplastin time (APTT) and prothrombin time (PT) are widely used to evaluate the intrinsic and extrinsic coagulation pathways, respectively [1, 2]. Despite their clinical importance, current coagulation testing systems face challenges in achieving rapid, reliable, and low-cost analysis, particularly in point-of-care settings.

Conventional coagulation detection techniques primarily rely on optical, mechanical, and acoustic methods. Optical detection, such as photometric clotting assays [3], measures light transmission or scattering to quantify clot formation. Mechanical and acoustic methods monitor viscoelastic changes in blood samples during coagulation [4, 5]. Although these techniques provide accurate measurements, they often require bulky instrumentation, complex operations, and are susceptible to environmental interference such as bubbles and particulate contamination [6, 7].

In contrast, electrical and electrochemical detection methods have become a simpler and more adaptable alternative, attracting increasing attention in recent years. Electrical sensing strategies detect impedance or voltage variations to monitor clotting situations [8, 9]. These approaches enable direct signal acquisition, easier system integration, and reduced dependence on complex optical or mechanical components, making them particularly suitable for miniaturized and point-of-care applications.

However, translating these electrical sensing strategies into direct coagulation assays remains challenging, particularly in achieving real-time monitoring of dynamic clot formation without relying on additional labeling or complex signal interpretation. To address these limitations, triboelectric nanogenerators (TENGs) have emerged as a promising self-powered sensing approach capable of directly responding to fluid property changes, such as viscosity variations during coagulation [10–12].

TENGs generate voltage or current signals through the contact and separation of heterogeneous materials, making them inherently sensitive to viscosity and other fluid characteristics [13, 14]. Lin et al. [15] demonstrated that triboelectric charges generated by liquid flowing over a SiO₂ surface could detect solution ionic content and conductivity, indicating TENG’s potential for biochemical sensing. Similarly, Lin et al. [16] measured surface charge density changes of aqueous solutions to assess parameters such as temperature and NaCl concentration, showing TENG’s sensitivity to physicochemical variations. Fan et al. [17] designed a preconcentration TENG that manipulates ion distributions in microfluidic channels without external power, highlighting its suitability for portable, low-energy diagnostic devices.

Despite the increasing number of studies integrating triboelectricity and microfluidics for biomedical detection, most investigations have relied on model liquids or idealized samples. Only limited studies have employed real clinical specimens, such as blood or urine, which restricts the clinical relevance and translational potential of triboelectric-based sensing platforms. In our previous work, we addressed part of this gap by developing a triboelectric microfluidic nanosensor for point-of-care platelet quantification using real human plasma [18]. That system demonstrated that platelet adhesion to collagen-coated microchannel walls altered flow resistance, leading to measurable changes in triboelectric voltage as platelet concentration increased from platelet-poor plasma (PPP) to platelet-rich plasma (PRP). These findings confirmed that triboelectric signals are sufficiently sensitive to detect physiologically relevant changes in blood properties.

Building upon this foundation, we propose extending triboelectric sensing to direct assessment of blood coagulation. During clot formation, plasma viscosity continuously increases as fibrin networks develop, which in turn modulates the liquid–solid contact dynamics within a microchannel. Because triboelectric voltage generation depends strongly on interfacial interactions between the flowing liquid and the channel wall [11, 12], variations in viscosity are expected to induce corresponding changes in electrical output. This provides a feasible strategy for quantifying clinically relevant coagulation parameters such as PT and APTT.

However, reliable detection of coagulation dynamics critically depends on the precise initiation of the biochemical reaction, which requires rapid and homogeneous mixing of plasma and coagulation reagents. Insufficient mixing can lead to delayed or spatially non-uniform clot formation, resulting in inconsistent viscosity evolution and unstable triboelectric signals.

PT and APTT measurements typically occur within 12–45 s after reagent addition [14], and clot formation can initiate within less than one minute [19]. Therefore, rapid and efficient mixing of plasma, reagents, and calcium ions is essential prior to signal acquisition. To achieve this, micromixers are commonly integrated into microfluidic devices to enhance mixing performance under laminar flow conditions.

Micromixers are generally classified as active or passive. Although active strategies, such as pressure, electrokinetic, acoustic, and thermal, can enhance mixing, they require external energy input and increase system complexity, limiting their suitability for point-of-care applications. Passive micromixers are well-suited for microfluidic systems operating under low Reynolds number conditions due to their structural simplicity and ease of integration. Their mixing performance primarily relies on the generation of secondary flows [20], which arise from lateral forces perpendicular to the main flow [21, 22]. These flows are typically induced by geometric modifications of the channel, such as obstacles, expansion–contraction structures, or curved pathways that create velocity gradients between the inner and outer streamlines [23]. In curved channels, such velocity differences lead to the formation of symmetrical counter-rotating vortices, commonly referred to as Dean flows [24].

Various passive micromixer designs have been developed based on these principles [20]. Staggered herringbone structures can effectively enhance mixing by inducing chaotic advection [25], however, they often require relatively complex fabrication processes. In contrast, expansion–contraction geometries generate local shear gradients and perturb the velocity profile, offering a simpler and more fabrication-friendly approach to achieve efficient mixing [26, 27].

In addition to geometric perturbation, recent studies have increasingly employed curved microchannels to induce velocity differences between the inner and outer walls, generating secondary flow for passive mixing [24]. Typical designs include spiral and serpentine micromixers. While spiral mixers guide the flow toward a geometric center and are effective for standalone mixing, their layout is less favorable for integration with downstream functional modules [28]. In contrast, serpentine microchannels offer an extendable and flexible layout, making them more suitable for integration within multifunctional microfluidic platforms [29].

**Scheme 1:**
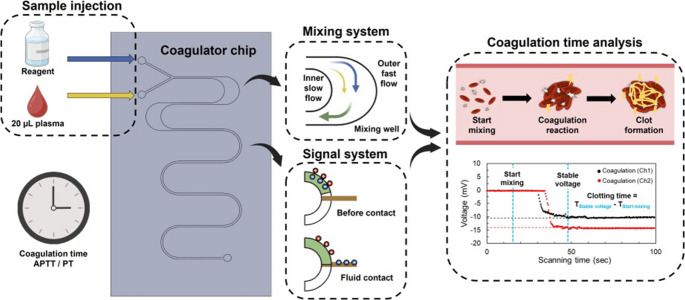
Schematic diagram of triboelectric sensor integrated microfluidic mixing system and triboelectric signal acquisition system for blood coagulation time measurement

In this study, we developed a microfluidic coagulator chip that integrates an expansion–contraction serpentine micromixer with triboelectric voltage sensing for real-time evaluation of blood coagulation, as shown in Scheme 1. The expansion–contraction serpentine microchannel was selected to balance mixing performance, fabrication simplicity, and system integration, while enabling rapid mixing within seconds through the combined effects of curvature-induced secondary flow and expansion–contraction-induced flow disturbance, which is well matched to the narrow PT/APTT detection window. Plasma samples and coagulation reagents are rapidly mixed within the microchannel, and the flowing liquid continuously interacts with the channel wall to generate measurable triboelectric signals collected by embedded copper electrodes. As clot formation progresses and plasma viscosity increases, the triboelectric voltage correspondingly changes, enabling the determination of PT and APTT. By combining efficient on-chip mixing with self-powered viscosity-sensitive detection, this platform provides a compact, low-cost, and rapid alternative to conventional coagulation analyzers, advancing the clinical applicability of triboelectric microfluidic systems for point-of-care blood monitoring.

## Experimental section

### Fabrication of the microfluidic device

**Fig. 1:**
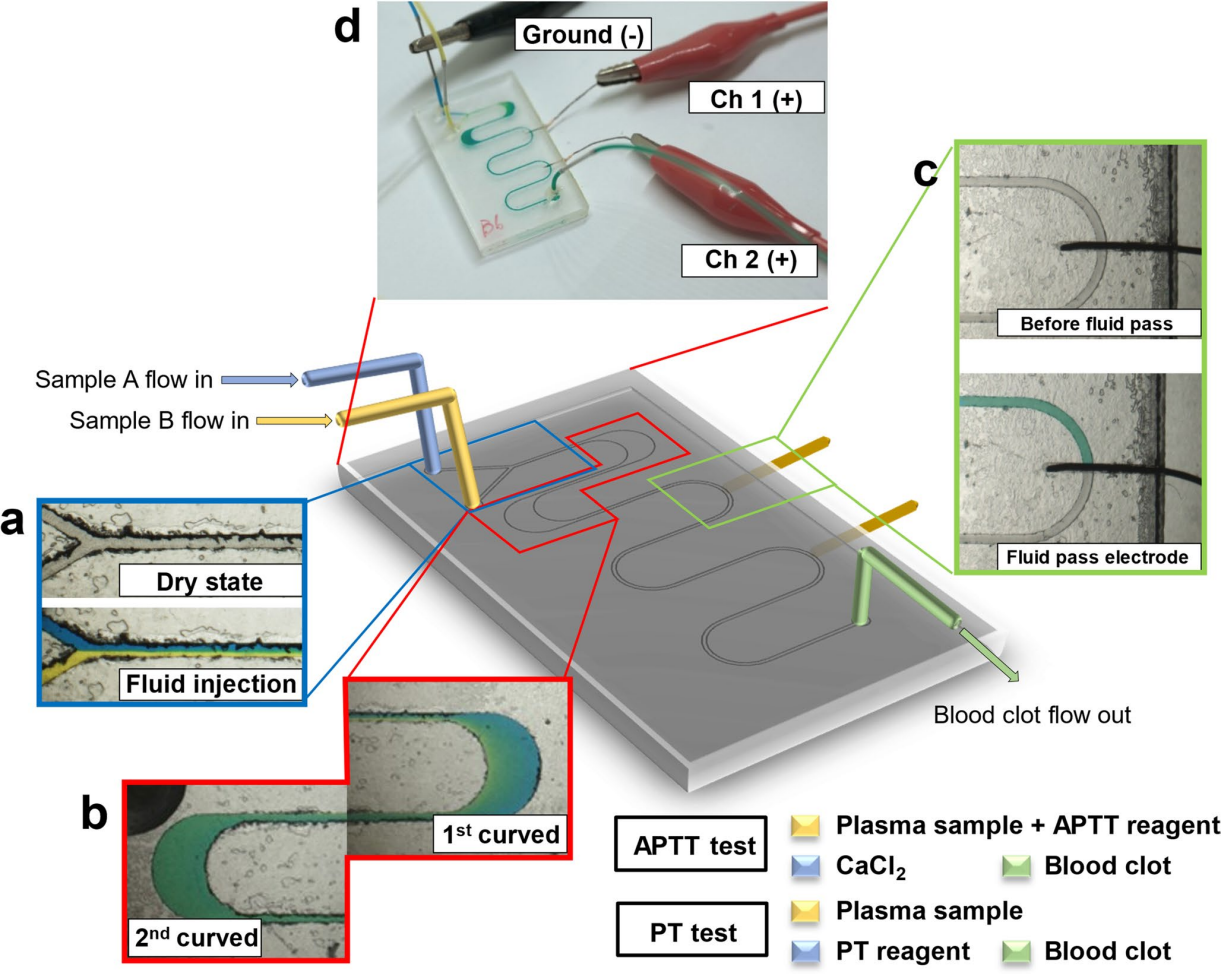
Schematic diagram of the coagulator chip. **a** Y-junction channel to gather two samples. **b** Two curves with an expansion and contraction cavity serpentine microchannel to mix the samples. **c** Copper electrode to collect the triboelectric signal. **d** Photo of the device and the electrical wires used to collect signals

In this study, a 2 mm thick polymethyl methacrylate (PMMA) was used as the substrate of the microfluidic device. First, a laser cutter (Universal VLS 2.30, USA) was used to cut the PMMA into a size of 5 cm × 9 cm. Then, a computer numerical control milling machine (Roland EGX-400, Japan) was used to make microfluidic channels and electrode grooves. A 0.25 mm milling cutter was used to engrave a microchannel structure with a width of 0.3 mm and a depth of 0.1 mm on the bottom plate with a specific pattern, as shown in Fig. 1. Electrode grooves with a width of 0.3 mm, a length of 6 mm, and a depth of 0.3 mm were milled on the top plate. The total device volume is approximately 10 µL, allowing complete microchannel filling to ensure reliable signal acquisition with minimal sample consumption.

Next, 0.3 mm diameter copper wires were embedded in the grooves as signal receivers at two designated positions, Ch 1 and Ch 2, corresponding to the first and second signal detection points in the microchannel, respectively. After the electrodes were embedded, the top and bottom PMMA plates were bonded together using a hot press (Cometech QC-677T, Taiwan) at a temperature of 60 °C and a pressure of 5 kgf/cm² for 3 min. After bonding is complete, the microfluidic device is finally assembled. Importantly, the microfluidic device was kept dry after fabrication and before sample introduction to ensure baseline consistency and avoid any premature reactions or fluid contamination.

### Establishment of the detection system

The system setup consists of three main parts: imaging, injection, and signal collection. For the mixing imaging part, to observe and analyze the mixing behavior inside the microfluidic channel, a stereo microscope (Motic SMZ-168, Hong Kong) was used in conjunction with an external CMOS camera (MIGHTEX SCE-C030-U, Canada). The CMOS camera was positioned to record the mixing area in real time to confirm the start mixing time and evaluate the mixing efficiency. We further analyzed it using ImageJ software (National Institutes of Health, Bethesda, MD, USA).

For the sample injection part, a dual-channel syringe pump (Harvard PHD 2000, Harvard Apparatus, USA) was used to inject fluids into the microfluidic device through two inlets. The pump was operated in a constant volume flow rate mode. This setting was chosen to facilitate the observation of changes in fluid viscosity because it allows the flow rate in the microchannel to respond to the viscosity of the fluid. This setting can apply a fixed mechanical pushing force based on the syringe size and its inlet-outlet connection to the microfluidic chip. Therefore, if the fluid viscosity changes, the actual flow velocity in the microchannel will also change, which affects the transition time to specific detection points such as Ch2. Therefore, at the same setting of volume flow rate, higher-viscosity fluids take longer to reach Ch2. In this study, we further validated this effect by testing fluids with different glycerol concentrations to demonstrate the impact of viscosity on transit time.

Last is for the signal acquisition part. The voltage signals were generated by the triboelectricity of the fluids between the channel walls, and were collected by a multimeter (GW Instek GDM-8261 A, New Taipei City, Taiwan). In addition, the multichannel scanning component (GW Instek GDM-8261 A scanner card) was connected to the multimeter and the copper wire electrodes of the microfluidic device. Then, each channel was connected to stainless-steel tubes at the inlet as a ground. After that, the real-time voltage signal could be scanned and recorded. By analyzing these signals, the corresponding voltage condition under different fluid properties can be determined.

### Micromixer channel design and mixing evaluation

The microfluidic channel was designed as an expansion–contraction serpentine structure to enhance mixing under low Reynolds number conditions. Based on the operating flow rate for APTT test is 20 µL/min and plasma properties (ρ ≈ 1025 kg/m³, µ ≈ 1.64 × 10⁻³ Pa·s), the Reynolds number (Re) in the straight microchannel was approximately 1.04, confirming laminar flow. In the expansion regions, Re further decreased to 0.13, indicating a strongly diffusion-limited regime. The corresponding Péclet number (Pe ≈ 10³) suggests that molecular diffusion alone is insufficient for effective mixing.

The serpentine curvature introduced secondary flow effects characterized by a Dean number (De ≈ 1.7), indicating the presence of weak Dean vortices that contribute to transverse mixing [30]. In addition, the expansion–contraction geometry induced significant variations in wall shear stress, ranging from ~ 1.09 Pa in contraction regions to ~ 0.11 Pa in expanded regions. This alternating shear field generates repeated stretching and folding of fluid interfaces, promoting chaotic advection and enhancing mixing efficiency.

To quantitatively evaluate mixing performance, the mixing index was calculated based on image intensity analysis following the method proposed by Fu et al. (2000) [31]. The mixing index is defined as:

$$\:{I}_{E}=1-\frac{1}{\stackrel{-}{I}}\sqrt{\frac{\sum\:_{i=1}^{N}{(I\left(i\right)-\stackrel{-}{I})}^{2}}{N}}$$

where $\:{I}_{E}\:$is the mixing index, $\:\stackrel{-}{I}$ is the average image intensity, $\:I\left(i\right)$ is the intensity of each pixel, and $\:N$ is the total number of pixels. A higher mixing index indicates more uniform mixing.

### Voltage signal definition and statistical analysis

Real-time voltage signals were recorded throughout each test and the relation between voltage and time was analyzed. Before sample injection, the microchannel was dry, with no triboelectric interaction, resulting in an initial balanced voltage. Once fluid contacted the electrodes, triboelectric charges were generated through solid–liquid friction, producing a clear voltage drop. As the fluid continued to flow, the voltage gradually stabilized and reached an endpoint. The endpoint was defined as the time when the voltage reached a plateau, so that the slope of the voltage change over time is nearly zero. Time shift refers to the delay between fluid contact with the electrode and the achievement of the endpoint, while voltage shift represents the change in voltage from the initial baseline to the stabilized value. Time shift and voltage shift are schematically illustrated in Fig. 3c.

To ensure reliability despite limited availability of real blood samples, each group of tests was repeated at least three times. Statistical analysis was performed using the standard deviation for each set of replicates as the error bar, representing data dispersion and enabling statistical inference. All measurements showed consistent trends with minimal variation. Furthermore, the electrical response was consistent with observed fibrin formation dynamics, demonstrating a correlation between voltage signals and the physical process of clot formation. Although the sample size was limited, the results demonstrated reproducible behavior across independent measurements, demonstrating the performance of the device.

### Plasma sample separation and preparation

Blood samples were from Shuang Ho Hospital, and their use was approved by the Taipei Medical University Joint Institutional Review Board (TMU-JIRB; no. N20230856). Blood samples were collected by the Vacutainer^®^ Citrate Tubes (Becton Dickinson (BD) Vacutainer^®^ Citrate Tubes 363080, Franklin Lakes, New Jersey, USA), which contained 3.2% sodium citrate as the anticoagulant, and blood and sodium citrate were mixed in a 9:1 ratio. Blood needs to be centrifuged within 1 h after it is drawn, and it was centrifuged at 1500 g for 15 min, and the upper supernatant was taken to obtain the anticoagulated plasma. Approximately 2 mL of whole blood was collected from each donor, and only 20 µL of plasma was used for each microfluidic test.

To simulate various clinical situations and validate the ability to detect different coagulation states, plasma from the same donor batch was subjected to serial adjustments in plasma proportion using phosphate-buffered saline (PBS) as the buffer. These samples with varying plasma fractions served as test samples to explore the detection range of the coagulator chip and its ability to respond to viscosity changes during the blood clotting. The 100% plasma sample represented undiluted plasma, while lower percentages indicated a smaller proportion of plasma in the test solution, with the remaining volume supplemented with PBS. Thus, a higher percentage corresponds to a higher contribution of plasma, which causes earlier clot formation. This approach allows the performance of the coagulator chip to be evaluated under a variety of coagulation conditions without causing variability in blood.

### APTT and PT tests with a commercial coagulator as a comparison

To validate the performance of the proposed coagulator chip, a commercial coagulator (TECO Coatron M2, Neufahrn, Germany) was used to detect specific coagulation times for comparison. We performed two common coagulation tests, APTT and PT, by the commercially available reagent kits for the test.

The liquid APTT reagent (TECO TEClot APTT-s A0320-050) lacks calcium chloride, necessitating the addition of extra calcium chloride during detection to initiate blood clotting. Specifically, 25 µL of anticoagulated plasma and 25 µL of the APTT reagent were warmed to 37 ℃, and well mixed in a test cuvette. After 3 min, 25 µL of 0.025 M calcium chloride was added to the cuvette, after which the blood immediately began to clot. The APTT result was obtained from the coagulator.

The PT reagent (TECO TEClot PT-s A0320-010) is a powder reagent, which must be reconstituted with 2 ml of pure water and pre-warmed to 37 ℃. Specifically, 25 µL of anticoagulated plasma was mixed with 50 µL of the PT reagent in the cuvette. The PT result was obtained from the coagulator.

### APTT and PT tests by the coagulator chip

To ensure compatibility with the commercial APTT and PT test procedure and to allow meaningful comparison with the microfluidic platform, the reagent preparation method was adapted for microfluidic injection.

According to the APTT kit instructions, the coagulation reaction begins immediately after the addition of calcium chloride to plasma that has been premixed with the APTT reagent. Therefore, in the microfluidic experiment, anticoagulated plasma premixed with the APTT reagent was used as one of the injected samples. This mixture was introduced through one inlet of the coagulator chip, and the calcium chloride solution was introduced simultaneously through another inlet.

For the PT test, calcium chloride is contained in the commercial PT kit, so anticoagulated plasma and the PT reagent were injected into separate channels of the coagulator chip at the same time. And when anticoagulated plasma gathers with PT reagent after the Y-junctional channel, the coagulation reaction immediately starts. These two designs of APTT and PT test in the coagulator chip ensured that the mixing and reaction within the chip were consistent with the APTT and PT protocol used in the commercially available device.

The microfluidic device recorded the instantaneous time and real-time voltage when the sample passed through each checkpoint. The clotting time was measured by the coagulator chip, which is determined by calculating the time difference between (1) the moment when the mixed sample reaches the Y-junction and (2) the moment when the voltage signal stabilizes, which means the clotting cascade is finished.

## Results and discussion

### Microfluidic mixing system

To confirm the mixing effect of the microfluidic device, water with blue and yellow dyes was used as samples in this study. The green image appeared when blue and yellow samples were mixed, as shown in Fig. S1.

Curves of the expansion and contraction cavity serpentine microchannel can evenly accelerate fluid mixing. The analytical results are shown in Fig. S1. The mixing indexes at checkpoints 2 and 4 were 0.92 and 0.97, respectively, which were both larger than 0.88 and 0.93 at checkpoints 1 and 3, respectively. Also, the mixing indexes at Ch 2 and Ch 3 were similar, which means that mixing was mainly caused by curves in the expansion and contraction cavity serpentine microchannel. In addition, the mixing effect could be increased by adding more curved channels to the design.

Water was injected into the microfluidic device at different flow rates to analyze the mixing effect. An image is shown in Fig. S2. When the flow rate increased, the boundary layer became apparent. This implies that the secondary flow effect generated by the curving channel was weakened, so the mixing effect was insufficient. When the flow rate increased to 10 and 15 µL/min, the boundary layer appeared at curves 1 and 2, respectively. In comparisons of the mixing index at each flow rate, when the mixing index was < 0.9 and < 0.85, a blurred and obvious boundary layer appeared in each instance. These results showed that the curves in the expansion and contraction cavity serpentine microchannel effectively reduced the impact of the flow rate on fluid mixing.

**Fig. 2:**
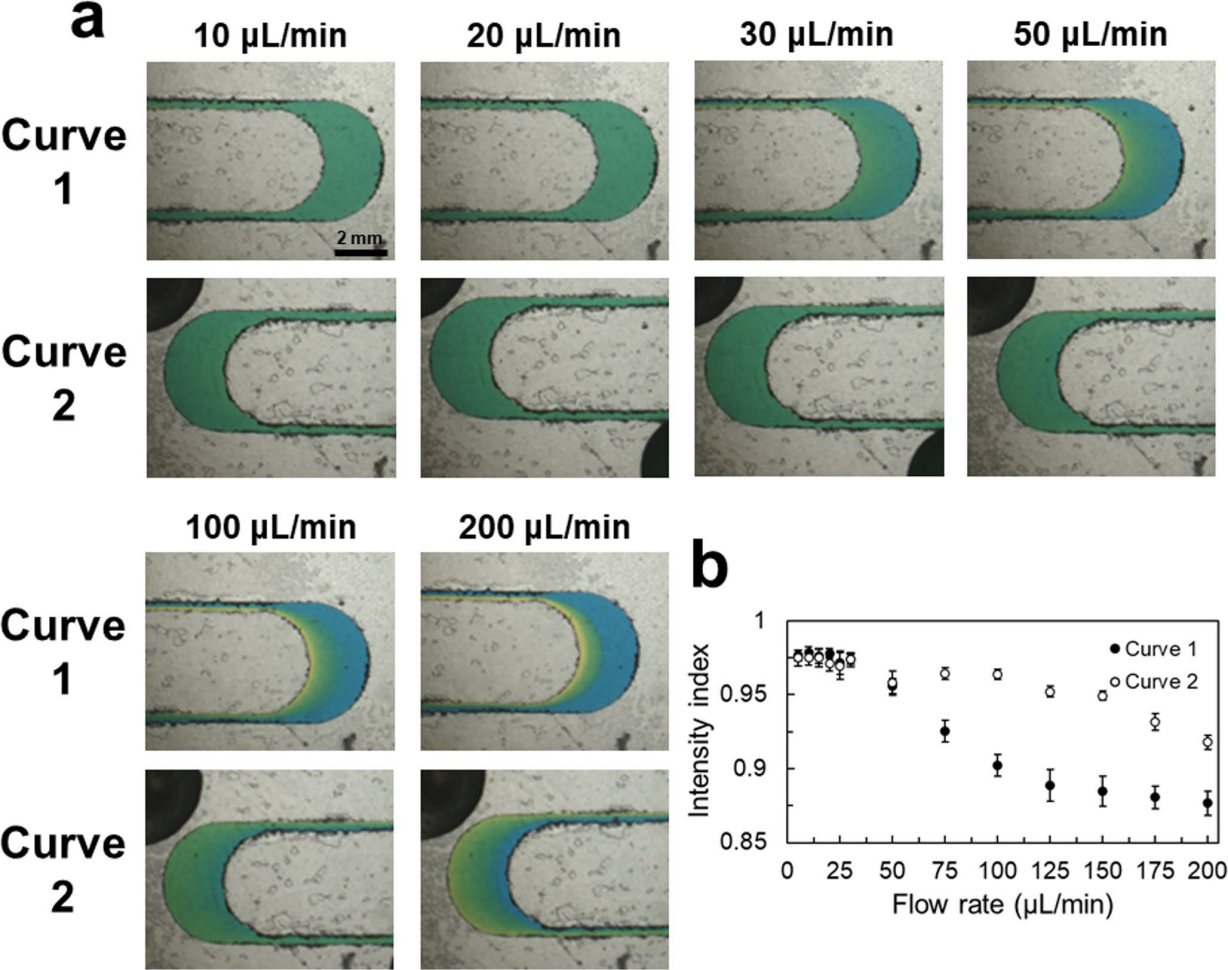
Mixing effect analysis of 20% glycerol and water. **a** Mixing images at curves 1 and 2 with different flow rates. The blue fluid is 20% glycerol, and the yellow fluid is water. **b** The mixing effect of different flow rates at curves 1 and 2

Glycerol at 20% with blue dye and water with yellow dye were injected into the microfluidic device to simulate the mixing effect of blood clotting. Images are shown in Figs. 2a and S3. When the flow rate increased to 30 µL/min, the fluids could still be evenly mixed in curve (1) Flow rates were increased to determine limitations of mixing in each situation. Images show that when the flow rate increased to 50 and 100 µL/min, a blurred boundary layer respectively appeared at curves 1 and (2) Therefore, the mixing index analysis is shown in Fig. 2b, and the flow rate limitation for even mixing for blood clotting was 100 µL/min, for which the mixing index was greater than 0.95. In addition, compared to the above sample situations, when the sample had a higher viscosity, it had a higher mixing effect at the same flow rates, and could also withstand higher flow rates. Importantly, under the operating condition used for coagulation measurement (20 µL/min), the mixing index exceeded 0.95 before the fluid reached the downstream detection region, indicating that plasma, reagents, and calcium ions were sufficiently mixed prior to the onset of clotting.

Time-resolved visualization of the mixing process was further conducted at a flow rate of 20 µL/min using 20% glycerol and deionized water, shown in Fig. S4, Fig S5, and Video S1. Images were acquired at 0.2 s intervals, focusing on the first and second curved regions of the serpentine channel. These observations clearly demonstrate rapid homogenization of the two fluids within a short time scale.

These findings suggest that the expansion–contraction serpentine design facilitates rapid on-chip mixing under the operating conditions. This is particularly important for coagulation assays, where sufficient mixing must be achieved within a limited time to ensure reliable measurement of PT and APTT.

### Signal acquisition system

**Fig. 3:**
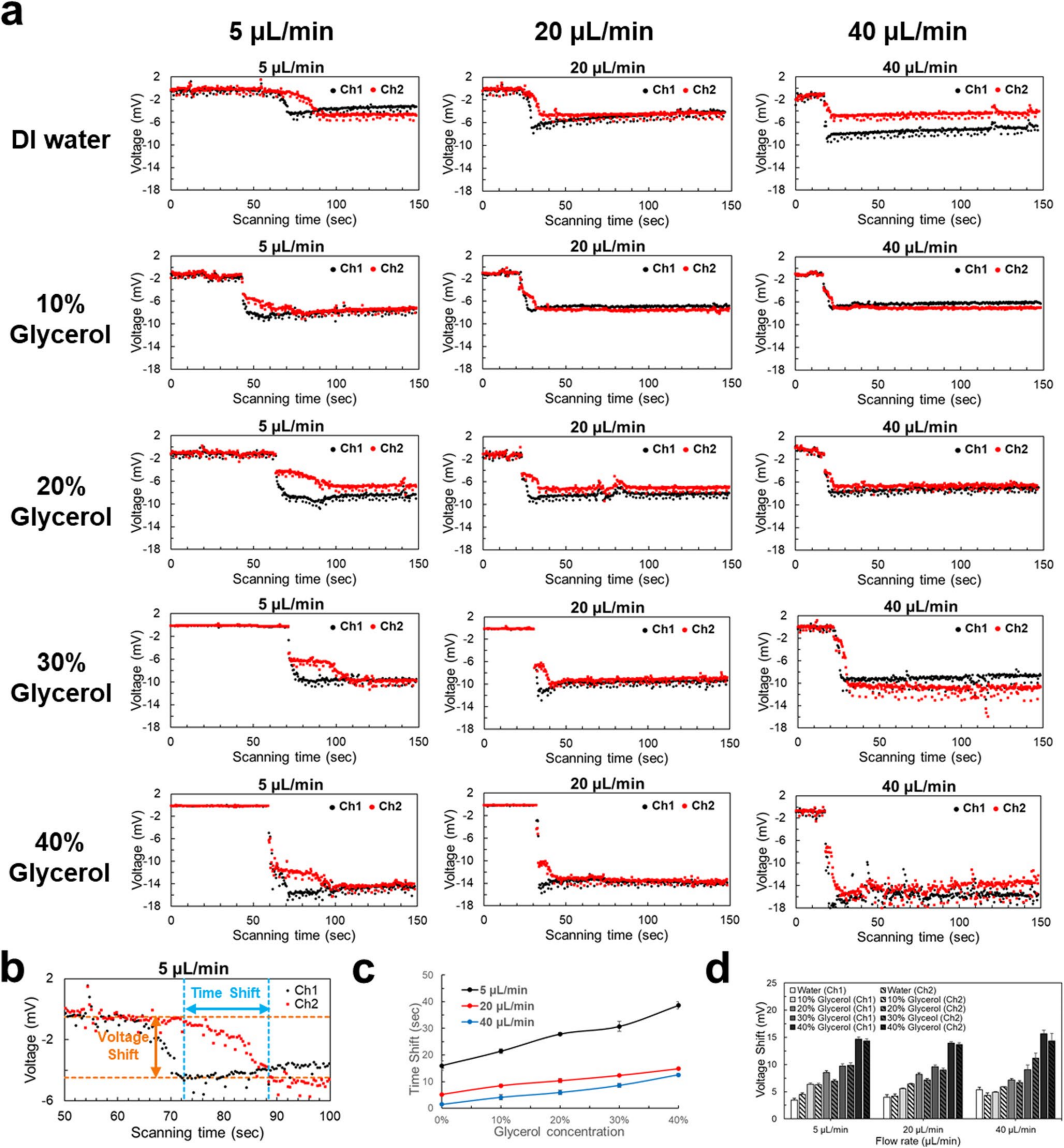
Time and voltage shift with different glycerol concentrations and water at different flow rates. **a** Real-time voltage signals at Ch1 and Ch2 for different flow rates and glycerol concentrations. **b** Label of the time shift and voltage shift. **c** Time shifts for different glycerol concentrations at 5, 20, and 40 µL/min. **d** Voltage shifts of Ch1 and Ch2 for different glycerol concentrations at 5, 20, and 40 µL/min. All tests were performed in triplicate (*n* = 3) to ensure result reproducibility

To confirm that voltage signals generated by the triboelectricity of the fluids between channel walls could be detected in the microfluidic device, water and 10% to 40% glycerol were separately injected into the microfluidic device at different flow rates. Voltage signals were collected through the copper wire electrodes at the corresponding curved channel positions of Ch1 and Ch2. Real-time voltage signals are shown in Fig. 3a.

Figure 3b shows time shifts of injecting water at 5 µL/min. Time shift reflects the time required for the signal to reach a stable state after fluid arrival at the electrode. The time shift analysis is shown in Fig. 3c. With a higher viscosity fluid, which means a higher concentration of glycerol, a longer time was required to pass Ch2 at the same flow rate. Also, with the same fluid, when the flow rate increased, the time shift of the fluid decreased because the fluid flows faster between the two detection points. Therefore, the time shift not only reflects the fluid flow’s properties in the microchannel, but also can serve as an indicator to qualify the fluid characteristics difference, especially in flow rate and viscosity.

Furthermore, significant voltage changes were detected when the fluid passed through Ch1 and Ch2, and this change gradually stabilized. The voltage shift shown in Fig. 3b and d reflect the voltage change from the initial balanced state, then through a voltage drop, and ending with voltage stability under each specific flow rate. The larger the voltage shift, the stronger the triboelectric effect between the fluid and the microchannel wall, which is mainly affected by the fluid viscosity.

A higher concentration of glycerol produced a larger voltage change, but the voltage changes did not significantly vary when the flow rate was in the range of 5 to 40 µL/min. This indicates that voltage variation is primarily governed by fluid viscosity rather than flow rate under the tested conditions. This behavior can be explained by the low Reynolds number in the system, where viscous forces dominate and inertial effects are negligible. Under such conditions, the electrical response is mainly influenced by fluid properties. Therefore, voltage variation provides a useful indicator for monitoring changes in fluid viscosity, such as those occurring during blood coagulation.

In addition, we observed an interesting phenomenon for high-viscosity fluids at Ch2, an initial voltage drop followed by a secondary voltage drop. This phenomenon may be due to incomplete mixing at the detection point. The first voltage drop is likely associated with the rapid release of accumulated charge when the fluid reaches the electrode, while the second voltage drop is more gradual, likely caused by the ongoing redistribution of charge during the mixing process. Nevertheless, the voltage eventually stabilizes so it can still capture the fluid states.

### APTT and PT measurement by the coagulator chip

To establish appropriate operating conditions for APTT and PT measurements, 20% glycerol, which has a viscosity comparable to that of plasma [32, 33], was used for the mixing and signal tests in the study. To further evaluate whether glycerol solutions can serve as a suitable surrogate, additional measurements were conducted using different glycerol concentrations, showing that the voltage response exhibited trends consistent with those observed in plasma samples, indicating that viscosity is the dominant factor influencing the triboelectric signal under the present conditions.

According to the coagulator manual, normal APTT and PT values for healthy people should be 27–42 and 12–18 s, respectively. When the clotting time is more than 300 s, the machine cannot detect the clotting time. Voltage signals of 20% glycerol in Fig. 3 show that when the flow rate was greater than 20 and 40 µL/min, the time it took for the voltage to stabilize was less than 27 and 12 s, respectively. The mixing images of 20% glycerol in Fig. 2 show that the fluid was evenly mixed when the flow rate was less than 100 µL/min.

Combining the results of voltage signals and mixing images, blood coagulation had the best detection ranges with flow rates of 20–100 and 40–100 µL/min, respectively, in the coagulator chip for the APTT and PT tests. A slower flow rate could be detected longer which made it easier to observe the blood coagulation situation, but required less blood to be consumed for detection. Therefore, 20 and 40 µL/min were respectively selected for the APTT and PT tests in this study.

### Optical and fluorescence validation of clotting process

The progression of clot formation within the microchannel was further examined using optical and fluorescence imaging, as shown in Fig. S6. Platelets were labeled using FITC anti human CD41 antibody (BioLegend, USA), emitting green fluorescence under 488 nm excitation, while fibrin was labeled using fibrinogen from human plasma, Alexa Fluor 647 conjugates (Thermo Fisher Scientific, USA), emitting far red fluorescence under 647 nm excitation. Fig. S6a shows the time dependent fluorescence images and their merged results reveal the progressive evolution of clot formation. Fig. S6b emerges from the fluorescence intensity analysis and indicates increasing accumulation of both platelets and fibrin over time, demonstrating the transition from a dispersed state to a structured clot.

To observe the mechanism of the clotting formation during the process, a magnified view of the fluorescence image at 30 min shows a dense fibrin network with characteristic fibrous structures, accompanied by platelet adhesion and aggregation along the fibrin strands, as shown in Fig. S6c. Bright field observation in Fig. S6d, further support these findings. Gradual clot formation was observed, with initial aggregation appearing at approximately 1 min and a consolidated clot structure forming around 3 min. These observations provide direct evidence of clot formation within the microchannel and support the interpretation of the corresponding electrical signals as indicators of coagulation dynamics.

### Blood clotting time measurement by the coagulator chip

To measure the blood clotting time by the coagulator chip, a real blood sample of the commercial APTT test was implemented. According to the kit instructions, anticoagulated plasma must be mixed with the APTT reagent first, and then the coagulation reaction occurs immediately when calcium chloride is added to the sample. To integrate this setup with the coagulator chip and ensure compatibility with a commercial coagulator, we premixed the anticoagulated plasma with the APTT reagent as one inlet sample and used Calcium chloride solution as the second inlet sample.

**Fig. 4:**
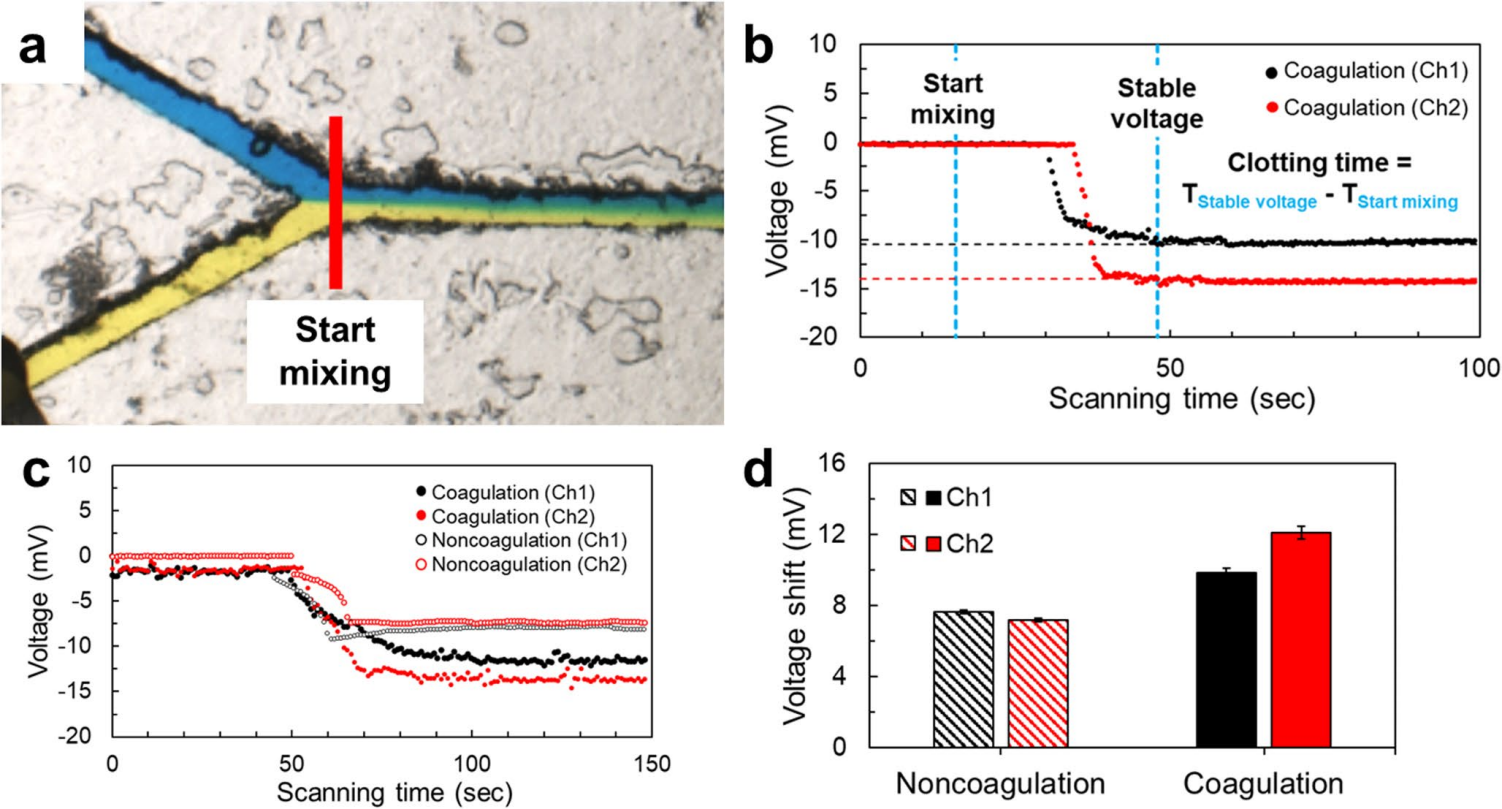
Blood clotting time measurement and qualification. **a** Observation of the Y-junction microchannel to define the start mixing time. **b** Clotting time measurement. **c** Clotting time compared to noncoagulation control. **d** Voltage shifts for the coagulation and anticoagulation groups

In the real-time voltage results shown in Fig. 4b, the starting time of the coagulation reaction was defined by the moment when the two fluids converged at the Y-shaped junction, as observed under a microscope and shown in Fig. 4a. The endpoint was determined by the time when voltage signals at both Ch1 and Ch2 reached a stable state. The clotting time detected by the coagulator chip was calculated as the time difference between these two points, as illustrated by the labeled lines in Fig. 4b.

To quantify the blood coagulation time by the coagulator chip, anticoagulated plasma and APTT reagents were mixed first, and then the calcium chloride solution or water was injected into the coagulator chip as a control of the coagulation and noncoagulation groups. As shown in Fig. 4c, the non-coagulation group exhibited rapid voltage decreases at both Ch1 and Ch2, with voltage shifts of approximately 7.5 mV, followed by signal stabilization after approximately 25 s, indicating that the fluid properties remained unchanged during the measurement.

In contrast, for the coagulation group, a rapid voltage drops also occurred, but the voltage shifts were larger than those for the noncoagulation group, and the voltage shifts were 10 and 12 mV at Ch1 and Ch2, respectively, as shown in Fig. 4d. This increase is associated with the conversion of fibrinogen into an insoluble fibrin network, accompanied by progressive clot formation and an increase in fluid viscosity. The larger voltage variation observed at Ch2 compared to Ch1 suggests that the fluid continued to undergo physicochemical changes along the channel, consistent with the ongoing coagulation process. Furthermore, the control experiment using anticoagulated plasma indicates that the influence of other plasma constituents, such as proteins and electrolytes, on the triboelectric signal is comparatively minor, with the signal primarily governed by viscosity changes under the present experimental conditions.

Compared to the blood coagulation test results, the clotting time for the commercial coagulator was 42.2 s, and for the coagulator chip developed in this study, it was 40.7 s. The difference between the coagulator chip and the coagulator was 3.6%, demonstrating high accuracy and practical applicability. In addition, by simultaneously monitoring Ch1 and Ch2, the coagulator chip can reveal the temporal and spatial changes in viscosity within the microchannel, further verifying its potential for real-time blood coagulation monitoring based on the triboelectric effect.

### APTT and PT test results with real plasma sample

Different plasma dilutions were prepared for the APTT and PT tests to test the limits of detection of the coagulator chip. To reduce variability caused by different patient blood sources, all diluted samples were derived from the same batch of plasma. By preparing dilutions from a single source, we ensured that any observed changes in signal or coagulation time were due to concentration differences rather than individual biological variability. PBS was used as the diluting buffer to prepare various plasma concentrations. The 100% plasma condition represented undiluted plasma, while other concentrations indicated the proportion of plasma present in the sample, with the remaining volume made up of PBS.

**Fig. 5:**
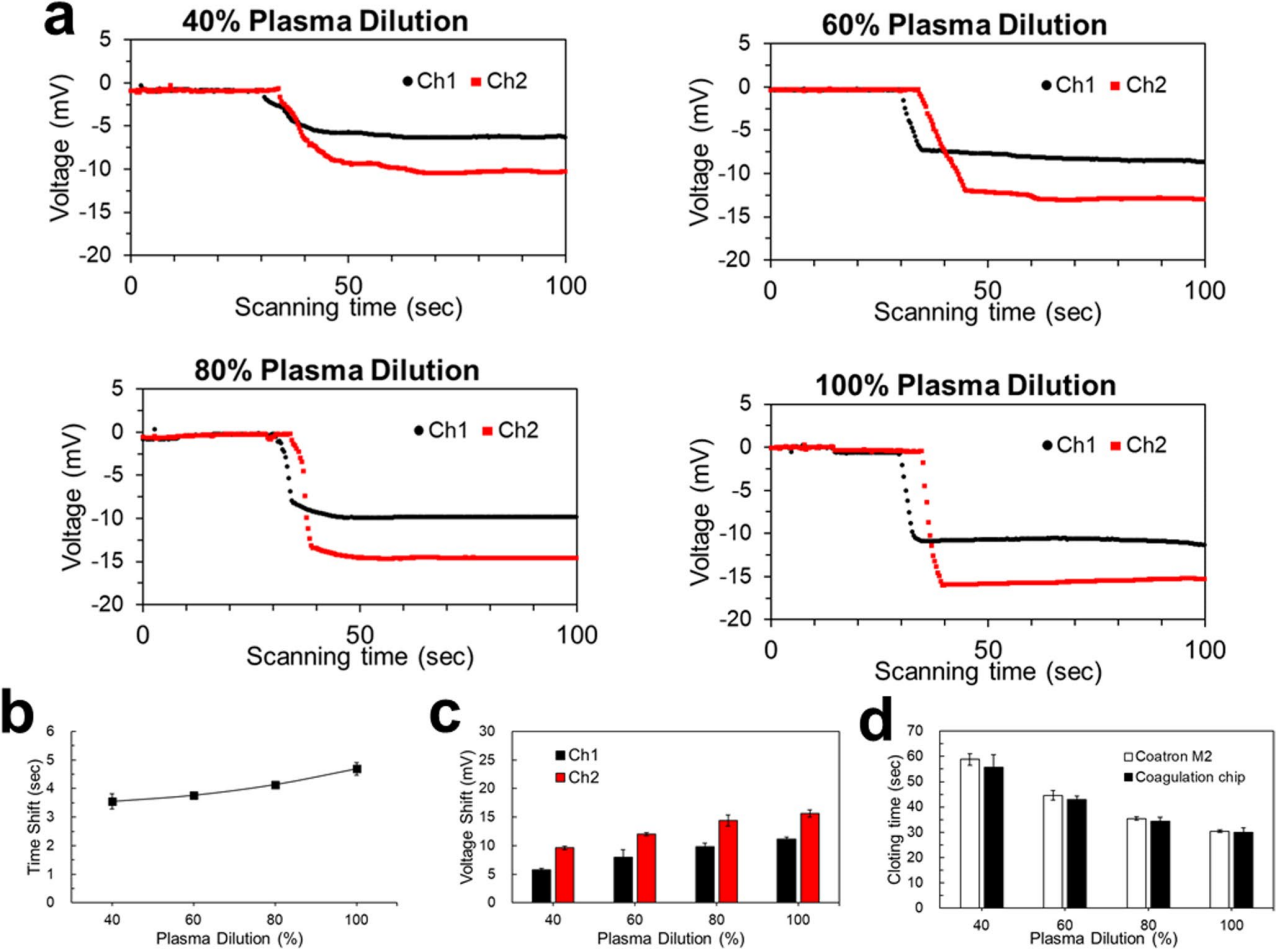
Activated partial thromboplastin time (APTT) test results. **a** Real-time voltage signals of the APTT test for 40% to 100% plasma dilutions. **b** Time shifts of different plasma dilutions. **c** Voltage shifts of different plasma dilutions. **d** Clotting time comparisons between the coagulator chip and a commercial coagulator, Coatron M2, of different plasma dilutions. All tests were performed in triplicate (*n* = 3) to ensure result reproducibility

APTT test results in Fig. 5 and S7 show that the time shift increased with plasma dilution, from 3.6 (40%) to 4.7 s (100%) as shown in Fig. 5b. Voltage shifts also increased from 9.59 (40%) to 15.62 mV (100%) as shown in Fig. 5c. Clotting time refers to the time point from the start of mixing to a stable voltage. Therefore, the clotting time decreased from 55.7 (40%) to 30 s (100%) as shown in Fig. 5d and Table S1. These results show that a higher plasma dilution caused the blood clot to form earlier, the flow rate to decrease, and the time shift to increase. In addition, a higher plasma dilution caused larger blood clots, so higher voltage signals were generated during the blood coagulation process.

**Fig. 6:**
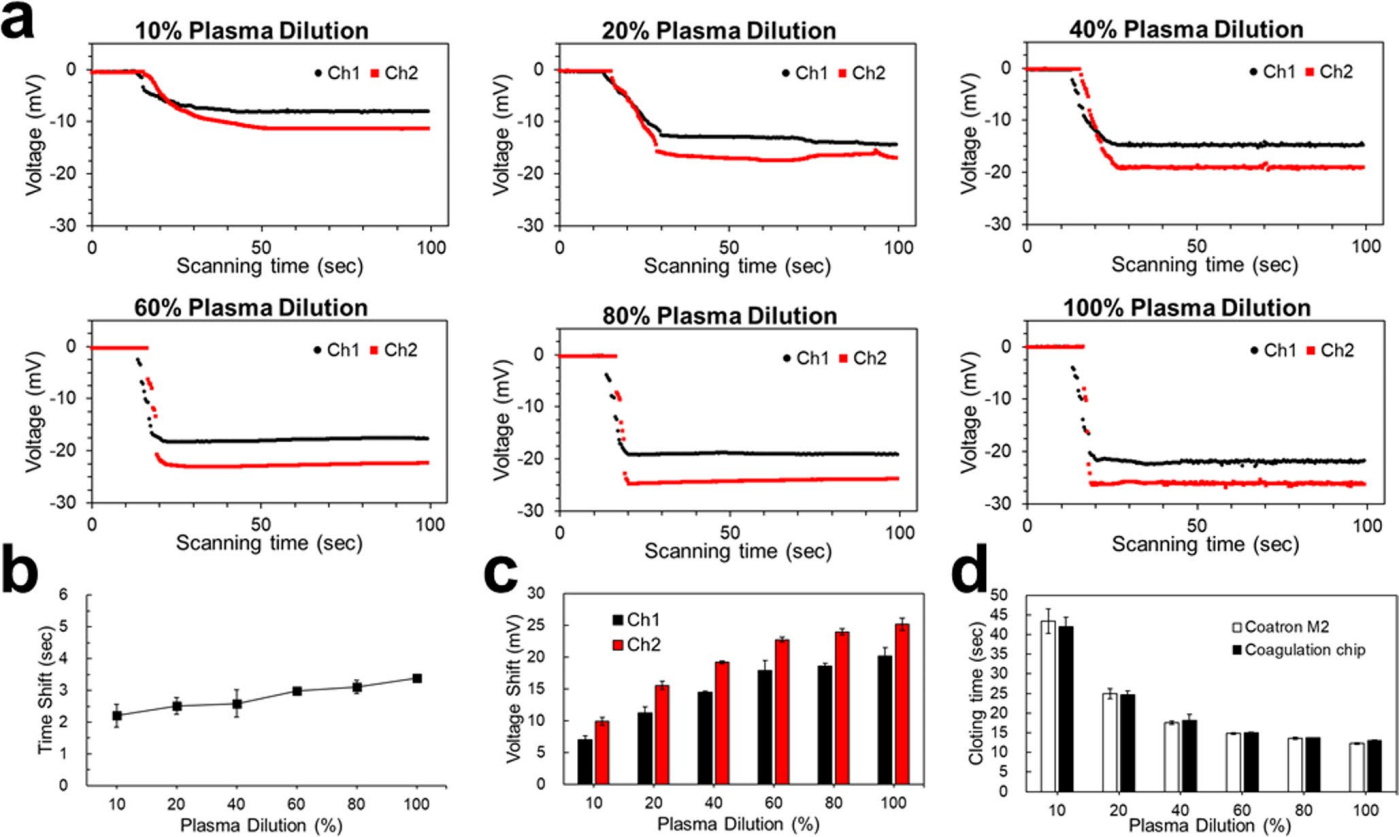
Prothrombin time (PT) test results. **a** Real-time voltage signals of the PT test for 10% to 100% plasma dilutions. **b** Time shifts of different plasma dilutions. **c** Voltage shifts of different plasma dilutions. (d) Clotting time comparisons between the coagulator chip and a commercial coagulator, Coatron M2, of different plasma dilutions. All tests were performed in triplicate (*n* = 3) to ensure result reproducibility

PTs had the same tendency as APTTs, and PT test results are shown in Fig. 6 and S8. However, due to different blood cascades, PTs had a larger detection range than APTTs. Time shifts of PTs increased with plasma dilution as shown in Fig. 6b. Voltage shifts also increased from 9.92 mV (10%) in 10% plasma to 25.17 mV (100%) as the plasma concentration increased, as shown in Fig. 6c. The clotting time decreased from 42.04 (10%) to 13.00 s (100%), as shown in Fig. 6d and Table S2.

To evaluate the performance of our microfluidic device in measuring blood coagulation time, we conducted parallel tests using the same plasma samples on both our coagulator chip and the commercially available TECO Coatron M2 coagulation analyzer. This approach ensured a direct comparison between our novel method and an established clinical standard.​.

The TECO Coatron M2 is an optical coagulation analyzer capable of performing various assays, including PT and APTT, with high precision and throughput, so it is usually used in the lab for coagulation studies. It utilizes an approved clotting algorithm with biphasic waveform analysis and features an autosense optics system. In our comparative study, we prepared plasma samples with varying dilutions and performed both APTT and PT tests. The results obtained from our device showed a high degree of correlation with those from the Coatron M2, with differences within approximately 5% and shown in Figs. 5d and 6d, Table S1, and Table S2. This level of agreement aligns with findings from previous studies comparing different coagulation analyzers, where consistent results were observed across devices.

These findings suggest that our coagulator chip can serve as a reliable and convenient alternative for measuring coagulation time, offering comparable accuracy to established commercial analyzers.

### Comparison of the device to the commercially available coagulator

In addition, practical considerations regarding device stability, cost, and operation were further evaluated. Due to the inherent nature of blood coagulation, fibrin formation may lead to channel blockage and affect repeated measurements. To ensure reliable performance and eliminate risks of cross-contamination and biofouling from residual proteins or clots, the microfluidic chip is designed as a single use disposable device. This approach avoids the need for complex cleaning procedures and ensures consistent electrical signal responses across measurements.

The chip is primarily fabricated from acrylic materials and simply embedded electrodes, making it highly suitable for mass production with an estimated cost of less than 1 USD per unit. Although additional equipment such as a syringe pump and data acquisition system are required, the total setup cost can be maintained below approximately 3,000 USD, which is significantly lower than conventional coagulation analyzers (typically 20,000–50,000 USD for clinical systems and 3,000–20,000 USD for smaller commercial instruments).

Regarding sample preparation, plasma separation is still required to ensure measurement accuracy, as whole blood contains various components that may interfere with electrical signal detection. This requirement is consistent with many commercial optical coagulation analyzers, which also rely on plasma samples. Nevertheless, the proposed device integrates rapid microfluidic mixing and real-time electrical detection, enabling fast coagulation analysis within seconds after sample preparation, thereby demonstrating strong potential for rapid testing applications.

Compared to conventional systems, the proposed microfluidic device offers advantages including reduced sample consumption, lower cost per test, and simplified operation workflow, while maintaining comparable measurement accuracy.

### Mechanism of the triboelectric effect in the coagulator chip

The triboelectric signal generation in the coagulation chip in this study is based on a fundamental physical mechanism, Solid-liquid triboelectric nanogenerators (SL-TENGs) [7, 12]. The triboelectric charge will generate when the fluid flows through the microchannel wall, and it will be collected through a signal acquisition electrode. Initially, the entire microchannel system is in a dry state before any sample injection. Therefore, the initial balance state is almost zero, as no liquid is present to generate or carry charges.

Once the fluid is injected into the channel, it begins to frictionally interact with the channel walls. These interactions lead to the generation of triboelectric charges due to solid-liquid contact electrification. The moving fluid then carries these induced charges downstream, and when the fluid front reaches specific signal acquisition sites, copper wire electrodes embedded at these locations extract the accumulated charges. This charge transfer induces an instantaneous voltage change, allowing real-time monitoring of the triboelectric voltage.

If the fluid properties remain constant, like the fluid with fixed fluid properties or noncoagulation plasma, this voltage change will display an initial sharp drop and then rapidly stabilize. This reflects the fact that in a laminar flow condition with a constant flow rate and fixed viscosity, the induced charge generation remains consistent over time. In such cases, once the initial redistribution of charge occurs at the electrode, no further changes in voltage are observed, and can define the fluid properties fixed at that situation.

To further interpret the relationship between fluid properties and electrical signals, a theoretical framework linking flow behavior and triboelectric output in the laminar system is introduced. According to the previous research from Li et al. [10], under low Reynolds number conditions, the effective flow behavior in a microchannel can be described by the relationship expressed as:

$$\:\mathrm{f}=\frac{32{{\upmu\:}}_{f}(a+b)}{{\uprho\:}{f}_{c}{V}_{pump}}$$

where $\:\mathrm{f}$ represents the channel resistance, $\:{f}_{c}$​ is the coefficient of channel resistance, $\:a$ and $\:b$ are the characteristic dimensions of the channel cross section, $\:{{\upmu\:}}_{f}$​ is the dynamic viscosity of the fluid, $\:{\uprho\:}$ is the fluid density, and $\:{V}_{pump}$​ is the imposed volumetric flow rate from the syringe pump.

Under laminar flow conditions, the channel resistance is primarily governed by the fluid viscosity. An increase in viscosity leads to higher hydrodynamic resistance, which in turn alters the effective flow behavior within the microchannel. Since triboelectric charge generation is closely associated with flow-induced interfacial interactions, the output voltage is therefore correlated with the fluid velocity. Consequently, viscosity evolution during coagulation directly influences the measured triboelectric signal. This interpretation is consistent with previous findings of the fluid and solid triboelectric generation mechanisms.

To ensure consistent and controlled flow conditions, all experiments were conducted using a syringe pump operating in constant volume flow rate mode. This mode ensures that the driving force for pushing the fluid through the microchannel remains stable across different tests and provides a controlled input rather than guaranteeing a strictly constant flow velocity within the microchannel. During coagulation, the formation of fibrin networks increases fluid viscosity, leading to higher hydrodynamic resistance. As a result, the actual flow velocity decreases, and the transit time required for the fluid to reach Ch2 increases.

Therefore, when a coagulating plasma sample is introduced, the dynamic increase in viscosity alters both the flow behavior and the triboelectric signal. By observing the time evolution of voltage signals at Ch1 and Ch2, the dynamic changes in fluid viscosity can be captured, and the real-time status of the coagulation process can be determined.

Both the theoretical framework and experimental results demonstrate that the triboelectric signal reflects the instantaneous physical properties of the fluid, particularly viscosity, enabling real-time monitoring of blood coagulation. This mechanism establishes the potential of the coagulator chip as a low-cost, portable, and low-sample-consumption alternative to conventional clinical coagulation tests such as APTT and PT.

## Conclusions

In summary, a microfluidic coagulator chip integrating efficient fluid mixing and triboelectric sensing was developed for the determination of PT and APTT. The Y-junction configuration enabled separate introduction of plasma and reagents, while serpentine microchannels with expansion–contraction cavities induced counter-rotating Dean vortices to accelerate mixing prior to clot formation. Two integrated electrodes detected voltage signals generated as fluids passed through the sensing regions, and a multi-signal acquisition system allowed real-time monitoring and comparison of signal variations associated with viscosity changes during coagulation.

Using diluted plasma samples obtained from real blood, only requires 20µL of sample and complete coagulation detection within 2 min, and the chip successfully defined clotting times, showing measurement deviation of less than 5% with results from a commercial coagulator (TECO Coatron M2). These results demonstrate that the device achieves rapid, low-volume, and reliable coagulation analysis. Fabricated from PMMA and operating through an electrical detection strategy, the device provides advantages including low cost, low sample and power consumption, portability, rapid analysis, and real-time monitoring capability.

Although the current configuration requires anticoagulated plasma and was evaluated using samples from a single donor to ensure controlled experimental conditions, it may not fully capture inter-individual variability in coagulation factors. Future work involving testing plasma samples from multiple donors is needed to further validate the robustness and clinical applicability of the proposed system. Nevertheless, the proposed platform demonstrates promising potential as an alternative approach for routine coagulation testing and highlights the feasibility of applying electrical sensing technologies to broader blood analysis applications.

## Supplementary Information

Below is the link to the electronic supplementary material.

Supplementary Material 1

Supplementary Material 2

## Data Availability

Data will be made available on request.

## Declarations

### Clinical trial number

Clinical trial number: not applicable.
