# Supplementary material for: A viscosity-induced voltage response microfluidic triboelectric sensor for real-time monitoring of blood coagulation
Source: Mikrochim Acta. 2026 Apr 21;193(5):325. doi: 10.1007/s00604-026-08044-0 (PMC13095917; doi:10.1007/s00604-026-08044-0)

**Supplementary Information**

**A Viscosity-Induced Voltage Response Microfluidic Triboelectric Sensor for Real-Time Monitoring of Blood Coagulation**

by

Jia-Cheng Lin^a,b,+^ , I-Chang Su^b,c,+^, Yong-Kwang Tu^b,c^,

Ningappa Kumara Swamy^d^, Kuang-Chong Wu^a^, Horn-Jiunn Sheen^a,*^, Yu-Jui Fan^e,f **^

^a^Institute of Applied Mechanics, National Taiwan University

*1 Roosevelt Rd., Sec. 4, Taipei 10617, Taiwan*

^b^Taipei Neuroscience Institute, Taipei Medical University, Taipei 11031, Taiwan

^c^Department of Neurosurgery, Shuang Ho Hospital, Taipei Medical University, New Taipei City 23561, Taiwan

^d^*Department of chemistry, JSS Science and Technology University, Mysuru-570006, India*

^e^ Department of Mechanical Engineering, National Yang Ming Chiao Tung University, Hsinchu 300093, Taiwan


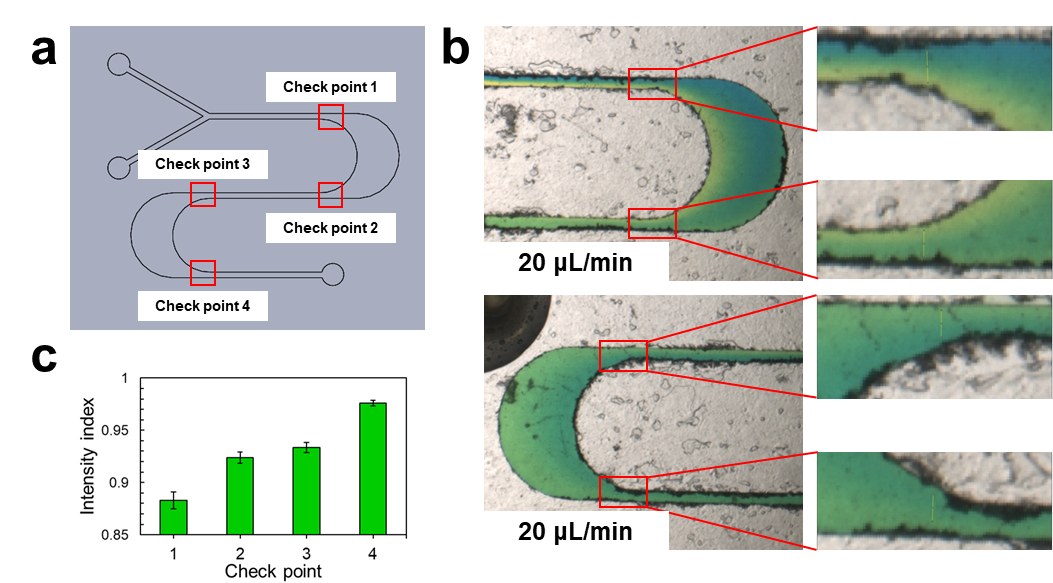


**Fig. S1**. Mixing effect test of the mixing part at curves with expansion and contraction cavity serpentine microchannel. (a) Check point of the mixing part. (b) Schematic diagram of image analysis. Inject water with blue and yellow dyes of 20 μL/min as a demonstration. (c) Mixing effect of each check point.


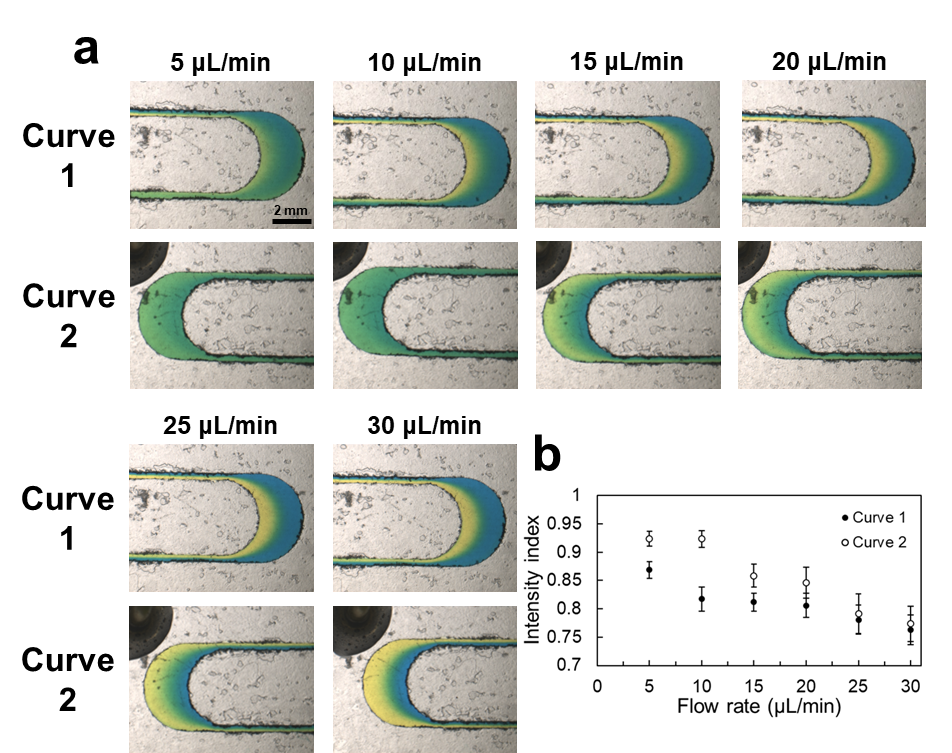


**Fig. S2.** Mixing effect analysis of water. (a) The mixing images at curve 1 and 2 with different flow rates. The blue fluid is 20% glycerol, and the yellow fluid is water. (b) The mixing effect of different flow rates at curve 1 and 2.


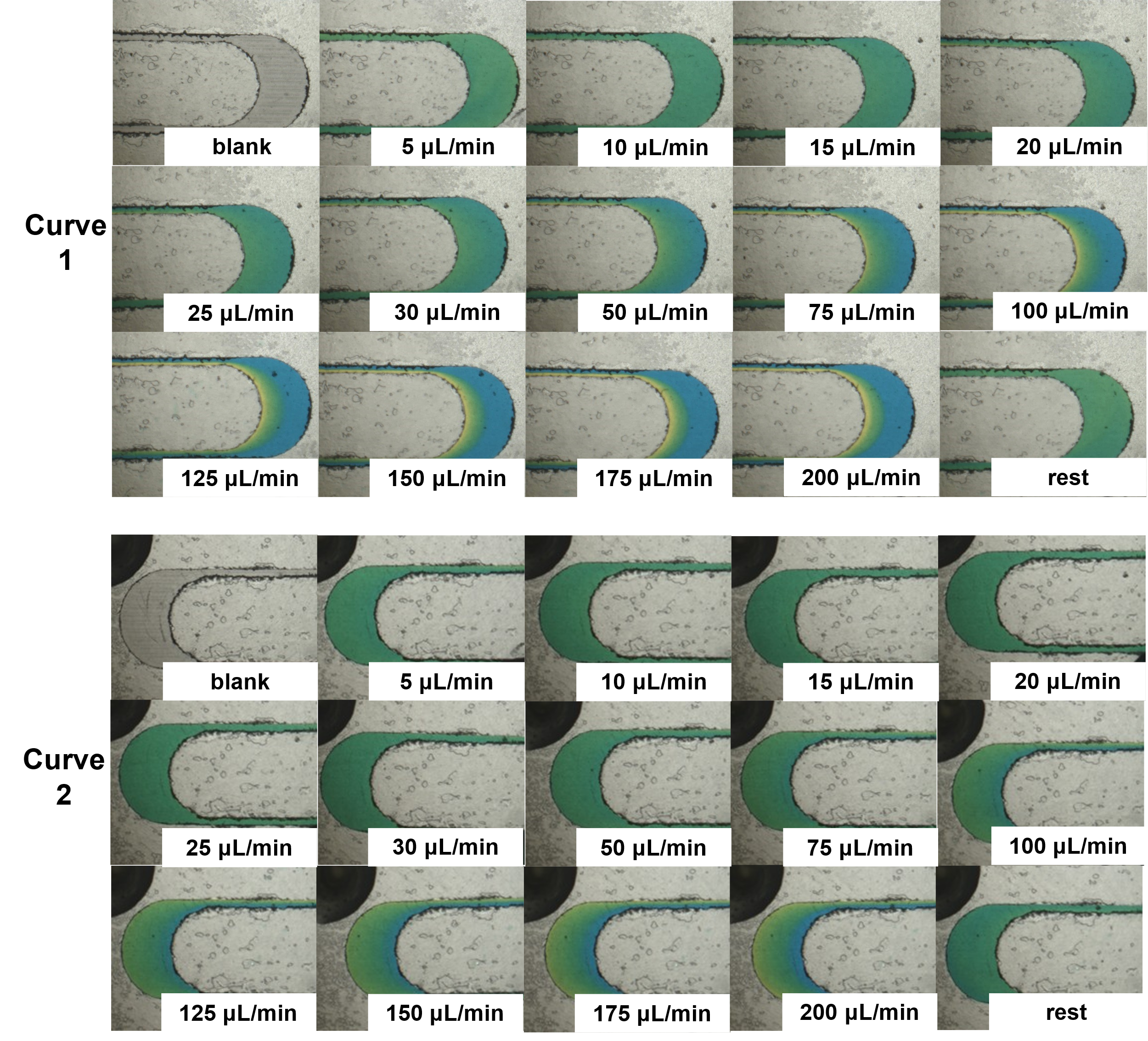


**Fig. S3.** Mixing image at curve 1 and curve 2 with different flow rates. The blue fluid is 20% glycerol, and the yellow fluid is water.


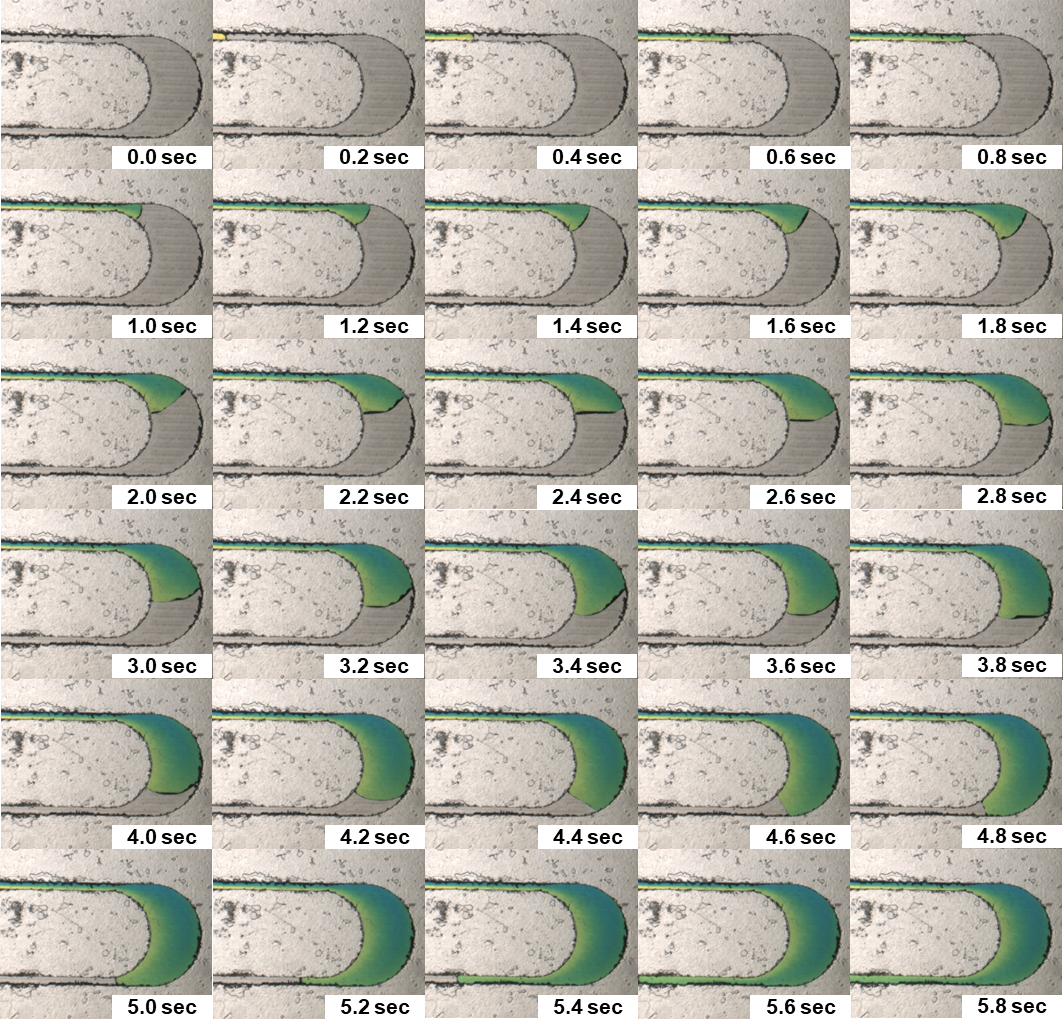


**Fig. S4.** Mixing image at curve 1 with the flow rate of 20 μL/min. The blue fluid is 20% glycerol, the yellow fluid is water, and the time interval is 0.2 second.


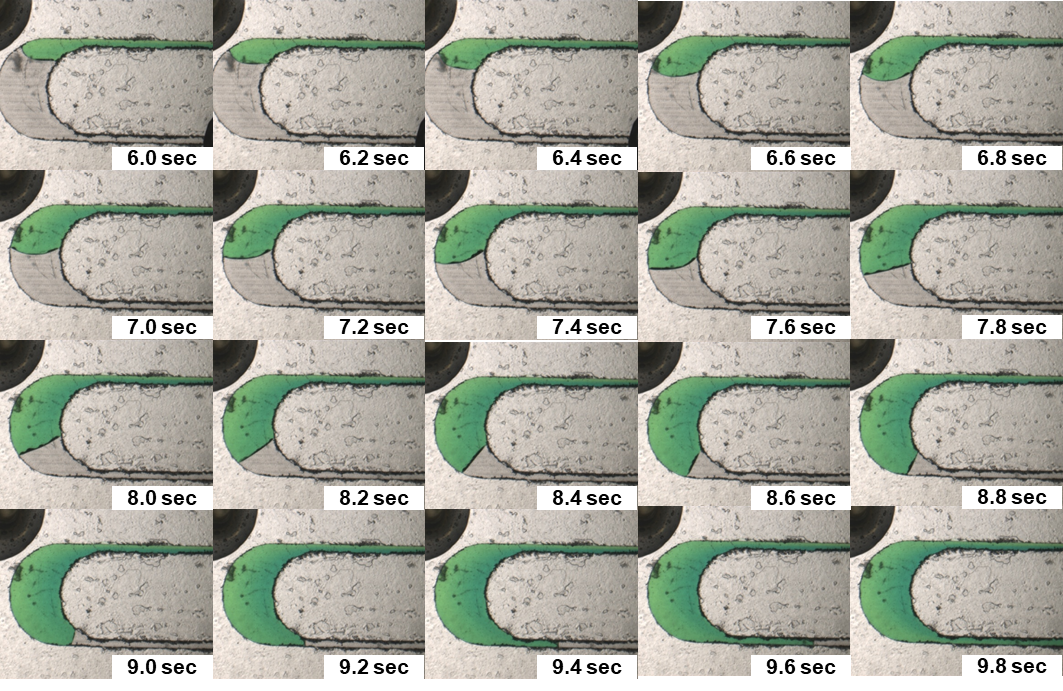


**Fig. S5.** Mixing image at curve 2 with the flow rate of 20 μL/min. The blue fluid is 20% glycerol, the yellow fluid is water, and the time interval is 0.2 second.


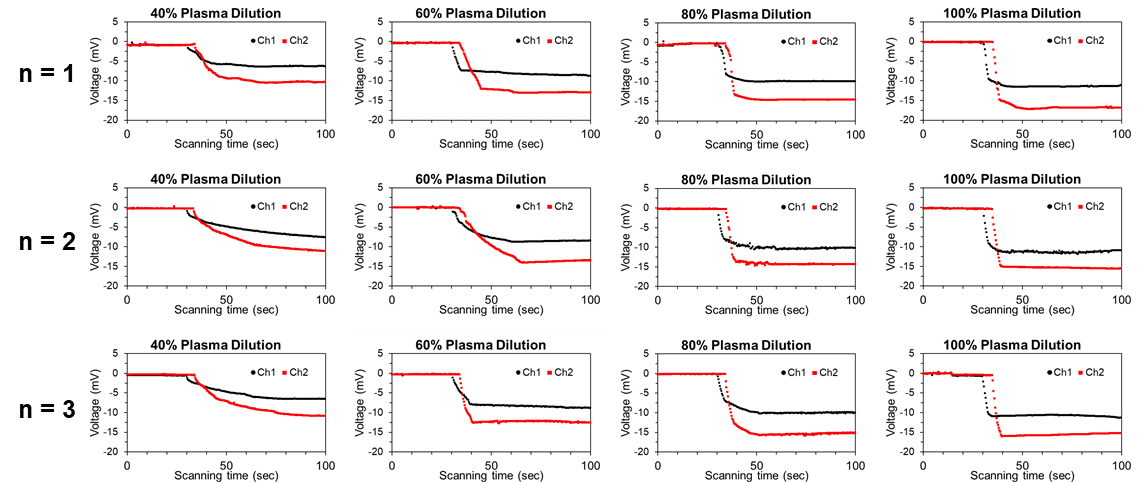


**Fig. S6.** Real-time voltage result of APTT test for different plasma dilution.

**Table S1.** The clotting time measured by the device and the APTT time test by coagulator under different plasma dilution.


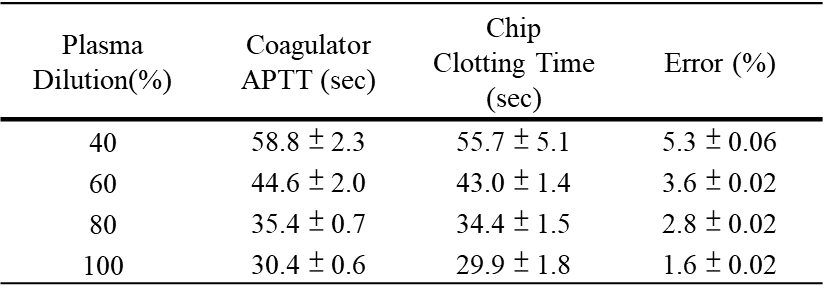


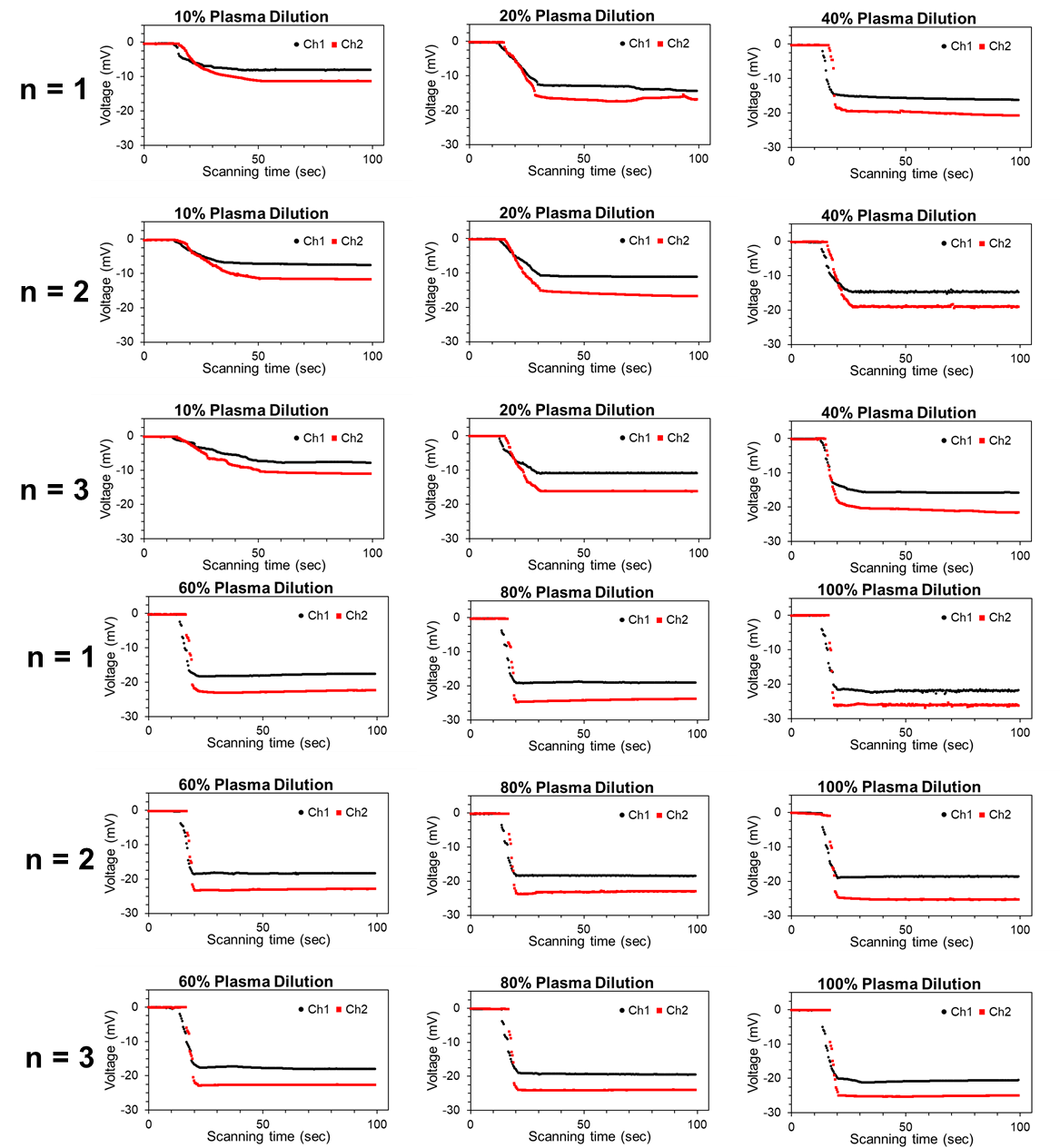


**Fig. S7.** Real-time voltage result of PT test for different plasma dilution.

**Table S2.** The clotting time measured by the device and the PT time test by coagulator under different plasma dilution.


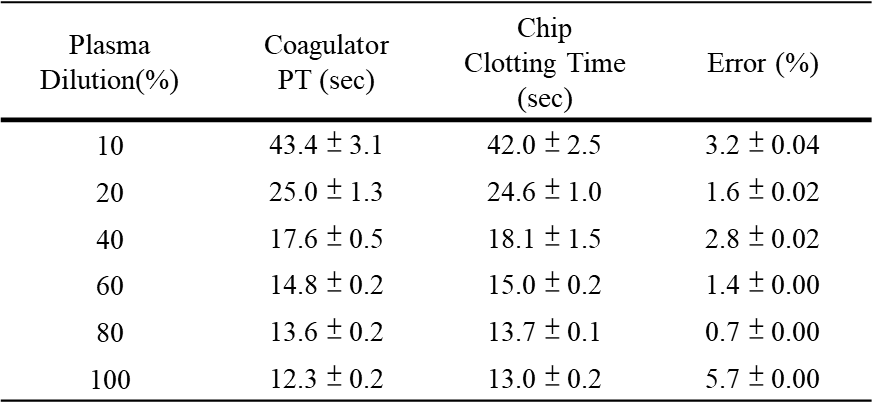

Supplement: Supplementary file 1 — Supplementary Material 1 [file 604_2026_8044_MOESM1_ESM.docx]
